# Prognostic value of subventricular zone involvement in relation to tumor volumes defined by fused MRI and O-(2-[^18^F]fluoroethyl)-L-tyrosine (FET) PET imaging in glioblastoma multiforme

**DOI:** 10.1186/s13014-019-1241-0

**Published:** 2019-03-04

**Authors:** Maciej Harat, Bogdan Małkowski, Krzysztof Roszkowski

**Affiliations:** 1 Department of Oncology and Brachytherapy, Nicolaus Copernicus University, Ludwik Rydygier Collegium Medicum, Romanowskiej 2 St, ,85-796 Bydgoszcz, Poland; 2 Department of Positron Emission Tomography and Molecular Imaging, Nicolaus Copernicus University, Ludwik Rydygier Collegium Medicum, Bydgoszcz, Poland; 3 Department of Oncology, Radiotherapy and Gynecologic Oncology, Faculty of Health Sciences, Nicolaus Copernicus University Toruń, Bydgoszcz, Poland; 4 Department of Radiotherapy, Unit of Radiosurgery and Radiotherapy of CNS, Franciszek Lukaszczyk Oncology Center, Bydgoszcz, Poland

**Keywords:** Glioblastoma multiforme, FET PET, Subventricular zone, Prognosis, Imaging biomarker

## Abstract

**Background:**

Subventricular zone (SVZ) involvement is associated with a dismal prognosis in patients with glioblastoma multiforme (GBM). Dual-time point (dtp) O-(2-[^18^F]fluoroethyl)-L-tyrosine (FET) PET/CT (PET) may be a time- and cost-effective alternative to dynamic FET PET, but its prognostic value, particularly with respect to SVZ involvement, is unknown.

**Methods:**

Thirty-five patients had two scans 5–15 and 50–60 min after i.v. FET injection to define tumor volumes and SVZ involvement before starting radiotherapy. Associations between clinical progression markers, MRI- and dtp FET PET-based tumor volumes, or SVZ involvement and progression-free (PFS) and overall survival (OS) were assessed in univariable and multivariable analyses.

**Results:**

The extent of resection was not related to outcomes. Albeit non-significant, dtp FET PET detected more SVZ infiltration than MRI (60% vs. 51%, *p* = 0.25) and was significantly associated with poor survival (*p* < 0.03), but PET-T1-Gad volumes were larger in this group (*p* < 0.002). Survival was shorter in patients with larger MRI tumor volumes, larger PET tumor volumes, and worse Karnofsky performance status (KPS), with fused PET-T1-Gad and KPS significant in multivariable analysis (*p* < 0.03). Uptake kinetics was not associated with treatment outcomes.

**Conclusions:**

FET PET-based tumor volumes may be useful for predicting worse prognosis glioblastoma. Although the presence of SVZ infiltration is linked to higher PET/MRI-based tumor volumes, the independent value of dtp FET PET parameters and SVZ infiltration as prognostic markers pre-irradiation has not been confirmed.

**Electronic supplementary material:**

The online version of this article (10.1186/s13014-019-1241-0) contains supplementary material, which is available to authorized users.

## Background

Glioblastoma multiforme (GBM) is the most aggressive malignant primary central nervous system tumor. While the majority of GBMs have similar pre-treatment magnetic resonance imaging (MRI) characteristics, subgroups exist with distinct clinical behaviors, genetic alterations, and outcomes. According to grading prognostic assessment (GPA) scoring, patients with newly diagnosed GBM qualifying for chemoradiotherapy have a two-year overall survival (OS) of between 5 and 35%, but 5–10% of GBM patients experience long-term survival [1]. Identifying prognostic groups who would benefit from different, personalized treatment remains challenging.

Age, Karnofsky performance status (KPS), and extent of surgery are all prognostic in GBM [2, 3], and more recently prognostic biomarkers have been described including O6-methylguanine-DNA methyltransferase (*MGMT*) promoter methylation [4, 5], isocitrate dehydrogenase 1 or 2 gene mutations [6, 7], and subventricular zone (SVZ) involvement [8]. However, the relationship between pre-irradiation MRI contrast enhancement-based tumor volume and clinical outcome remains controversial [9, 10].

Therefore, accurately predicting tumor behavior in individual patients based on imaging parameters remains challenging, especially when molecular-genetic factors are not available. Imaging may be especially important given that mutations show intratumoral heterogeneity in non-operable, sub-totally operated, or *MGMT* promoter status-undefined patients [11–13].

SVZ infiltration defined by MRI is known to be associated with treatment outcomes and progression and is thought to arise from neural stem cells [14, 15]. Extensive peritumoral edema on imaging may also be associated with survival [16, 17], since edema defined by MRI-T2 sequences may represent a mixture of neoplastic cells as well as vasogenic edema [18]. However, imaging parameters that more accurately define prognosis are still urgently needed to individualize treatment.

Positron-emission tomography/CT (PET) using O-(2-[^18^F]fluoroethyl)-L-tyrosine (FET) has been widely used for static and dynamic imaging in patients with brain tumors [19, 20]. Dynamic FET PET is helpful for defining aggression in WHO III astrocytomas [21] and low-grade gliomas (LGGs) [22, 23]. Moreover, WHO I-II gliomas show increased uptake kinetics compared to WHO III-IV high-grade gliomas (HGGs) [24, 25]. Dynamic acquisition more accurately differentiates LGGs from HGGs than standard static scans (20–40 min post-injection (p.i.)), mainly due to the characteristic high FET uptake in HGGs in the initial phase [26]. However, many institutions do not have routine access to dynamic PET imaging techniques. When dynamic PET cannot be performed, FET PET acquisition at a few selected time points may be a cost- and time-effective alternative as demonstrated using relatively early and very late time points (20–40 min p.i. and 70–90 min p.i.) [27]. However, experience with dtp FET and other amino acid PET tracers in patients with gliomas remains limited. Biological tumor volume defined by dtp FET PET correlates with progression site [28], but to the best of our knowledge, the prognostic impact of dtp FET PET parameters in GBM patients has yet to be determined. We hypothesized that dtp FET PET imaging in combination with SVZ infiltration would accurately select subgroups of patients with different chemoradiotherapy outcomes.

## Methods

### Study and patient details

This was a post-hoc analysis of a prospective study approved by the Ethics Committee of Collegium Medicum of Nicolaus Copernicus University (procedure nr KB257/2012), and all subjects signed written informed consent. Thirty-five consecutive patients with newly diagnosed GBM referred for radiotherapy planning between December 2012 and October 2014 and fulfilling pre-specified criteria were included. Inclusion criteria were: (i) KPS > 50 with normal mental status; age 18 years or greater; histopathological confirmation of GBM; previously untreated with radiation and/or chemotherapy; and time between PET examination and start of chemoradiotherapy no longer than 2 weeks. Patients underwent dtp FET PET scans at the Department of Nuclear Medicine, the Franciszek Lukaszczyk Oncology Centre in Bydgoszcz. Radiotherapy was performed in the Department of Radiotherapy, the Franciszek Lukaszczyk Oncology Centre in Bydgoszcz.

The maximum follow-up was 48 months. Progression-free survival (PFS) was measured from the start of radiotherapy to the date of tumor growth on conventional MRI according to Modified Response Assessment in Neuro-Oncology criteria [29]. All progressions were stratified into whether they occurred locally (within 2 cm of the primary tumor defined by MRI) or distantly (outside this margin). For survival analysis, family members were contacted to confirm the exact date of death. OS was defined as the time from the start of radiotherapy until death.

### MRI and 18F-FET PET/CT

All radiotherapy planning MRI studies were carried out using a Philips camera (3 Tesla; Achieva 3.0 T X-series, Philips Medical Systems, Crawley, UK) and a standard head coil up to 7 days prior to radiotherapy in two stages: (i) standard head MRI, taking the area containing tumor into account in the spin-echo or turbo spin-echo sequence in T1-, T2-, and PD-dependent images at three levels: frontal, sagittal, and transverse; and (ii) patients received intravenous contrast (gadolinium diethylenetriamine pentaacetate; Magnevist, Bayer Schering Pharma, Berlin, Germany) at a dose of 0.2 ml/kg body weight with 180 s of imaging using the spin-echo sequence in T1-weighted images in three dimensions. The scan thickness was 2 mm in a 512 × 512 pixel matrix. Tumor was defined as the area of contrast-induced signal enhancement in the T1 sequence. Hypointense areas without contrast enhancement on T1 images were regarded as the postoperative bed.

All PET/CT scans were performed using a mCT128 Biograph (Siemens Medical Solutions, Erlangen, Germany) using locally produced FET radiotracer. The amino acid ^18^F-FET was produced and applied as described previously [30].

Patients were fasted for 4 h prior to data collection. Radiotracer uptake was assessed after 5–15 min and 50–60 min after i.v. administration of 350 ± 10 MBq FET. Image acquisition was performed in the supine position after head immobilization with an individual thermoplastic mask fixed to the scanner table. CT scans were performed as follows: CARE Dose 4D, 120 kV, and pit 0.7 recorded every 2.7 min per 1 position of the bed. The TrueX+TOF (UltraHD-PET) three-dimensional algorithm was used for image reconstruction.

FET tissue uptake was recorded as a standardized uptake value (SUV) defined as the ratio of radioactivity (MBq/ml) of the tissue marker to the initial radioactivity of the marker administered i.v. according to the patient’s weight [31]. The tumor was assessed using the Leonardo™ diagnostic station (Siemens Medical Solutions/CTI).

To measure FET uptake, volumes of interest (VOI) were defined in similarly sized symmetrical areas defined by the tumor on one side and normal tissue in the other (normal) hemisphere. In the semi-quantitative analysis, 5–15 and 50–60 min after administering radiotracer, the maximum SUV (SUV_MAX_) and the mean SUV (SUV_MEAN_) were specified for each VOI on PET scans with CT images used as reference images. The SUV_MEAN_ and SUV_MAX_ ratios in the VOI of the tumor to healthy brain were determined (tumor-to-brain ratio, TBR_MAX_ and TBR_MEAN_). Tumors were contoured semi-automatically as areas corresponding to radiotracer uptake above 1.6 x SUV_MEAN_ in the VOI of normal brain (threshold) corrected to areas of physiological activity in the basal ganglia, thalamus, cerebellum, skull bones, sphenoidal sinus, sagittal sinus, pituitary, and vessels [31, 32]. The tumor area was defined this way 5–15 (PET_VOL_ 10) and 50–60 min (PET_VOL_ 60) after radiotracer administration. Fused volumes of the larger of PET and MRI volumes with (PET-T1-Gad) and without tumor bed (PET-T1-Gad without tumor bed) were assessed. A nuclear medicine specialist and radiation oncologist jointly evaluated each case.

### FET uptake values analysis

The differences between TBR_MEAN_10 and TBR_MEAN_60 (*TBR*_*MEAN*_ *diff*), TBR_MAX_10 and TBR_MAX_60 (*TBR*_*MAX*_ *diff*), SUV_MEAN_10 and SUV_MEAN_60 (*SUV*_*MEAN*_ *diff*), SUV_MAX_10 and SUV_MAX_60 (*SUV*_*MAX*_ *diff*) were calculated in each case. The difference between PET tumor volumes (*PET*_*VOL*_ *diff*) was also defined.

### Subventricular zone invasion and extensive peritumoral edema

**Fig. 1:**
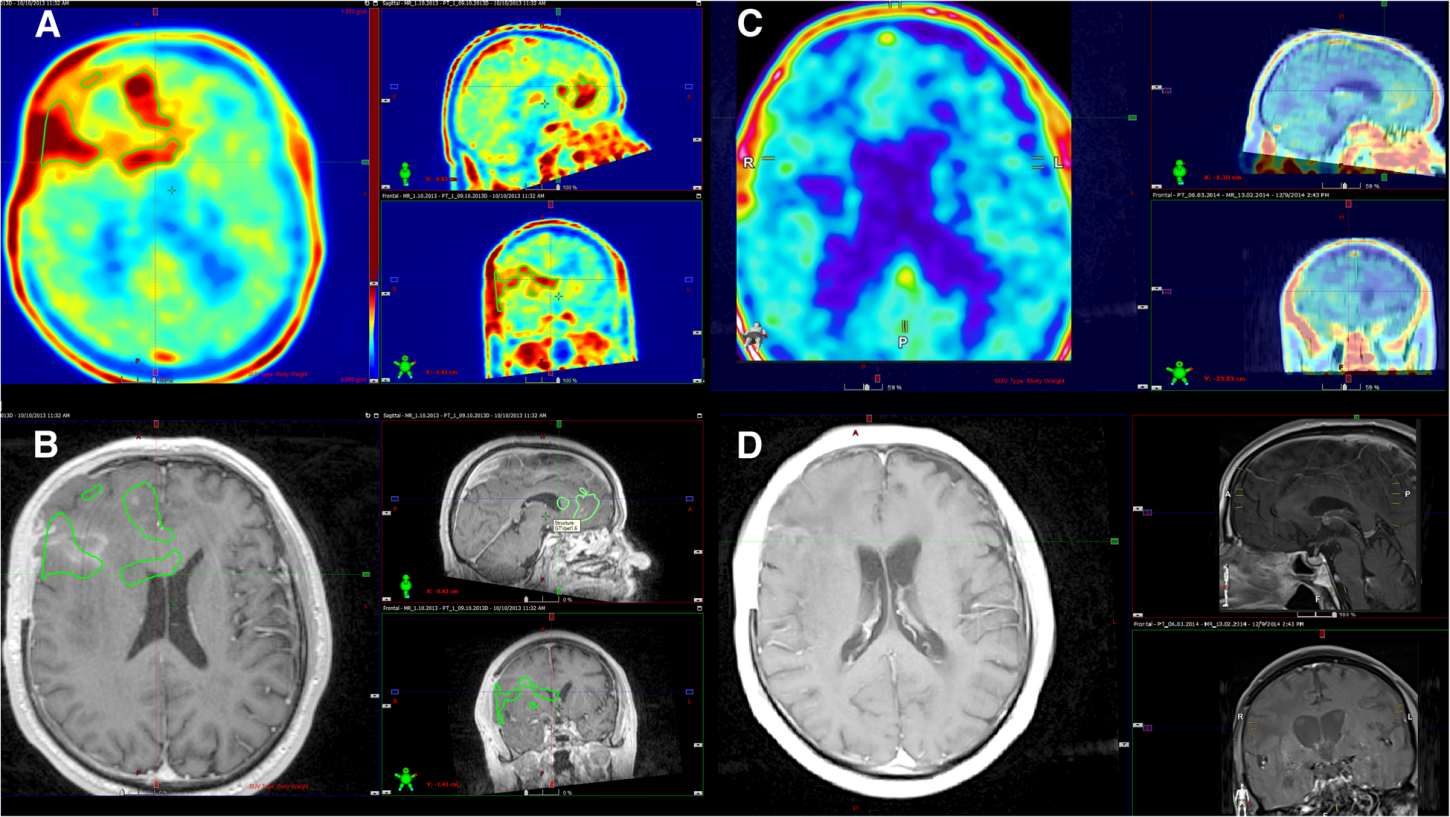
Comparison of pre- and post-irradiation dtp FET PET and MRI images of glioblastoma long-term survival in the right frontal lobe. Dtp FET PET volume < 40 cm^3^. The large pathological uptake volume extends into the SVZ over a substantial area (**a**). Contrast-enhanced T1-weighted MRI performed for radiotherapy planning with no SVZ infiltration in the axial, sagittal, and coronal planes (**b**). The patient had a favorable outcome, being alive at the end of the observation period (OS 47 months) without progression. Twelve months post-treatment dtp FET PET with complete response: TBR below 1.6 (**c**) and MRI with residual contrast enhancement (**d**)

SVZ was defined as contrast-enhanced lesions and/or dtp FET PET-positive uptake involving the wall of the lateral ventricle. Patients without SVZ involvement on MRI but infiltrated in PET were defined (Fig. 1).

Peritumoral edema was observed as hyperintense areas in T2-weighted or FLAIR MRI or hypointense areas in T1-weighted images. Extensive peritumoral edema (EPE) was defined when edema extended 2 cm from the tumor border as in [33]. SVZ and EPE were analyzed in relation to OS or PFS. Moreover, the OS and PFS of tumors involved SVZ (+) and not involved SVZ (−) in combination with all clinical and imaging parameters were analyzed. The median was used as the threshold for tumor volumes and imaging quantitative parameters.

### Statistical analysis

Calculations were performed in STATISTICA v13.0 (Statsoft, Poland). Quantitative parameters are presented as minimum and maximum values (min and max) and mean (□) and median values. Distributions were assessed using the Shapiro-Wilk test; parameters without a normal distribution were analyzed using the Mann-Whitney rank sum test. Spearman’s correlations were used to compare two quantitative parameters. For univariate analyses, Cox regression was used to assess the significance of individual variables using log-rank tests. OS and PFS were analyzed with Kaplan–Meier survival curves. The median was used as the threshold for dichotomizing parameters. To examine relative effects, multivariate regression analyses and log-rank (Mantel-Cox) testing were performed. *P*-values < 0.05 were considered significant.

## Results

### Overall characteristics

Thirty-five patients were eligible for study. During a mean observation period of 36 months, 32 patients (91%) died. The mean OS was 16 ± 2 months (range, 4–48 months), and the mean PFS was 10 ± 2 months (range, 2–47 months). The clinical parameters including MRI and PET tumor volumes are summarized in Table 1.

**Table 1:** Clinical and radiological parameters of the study population

	*n* (range)	%
Gross total resection defined with MRI	24	68
KPS performance > 70	24	68
Age (mean)	53 (29–73)	–
Sex (male)	25	71
SVZ infiltration defined by MRI	18	51
SVZ infiltration defined by dtp FET PET	21	60
EPE	10	28
Distant progression	9	25
TBR_MAX_ increased	9	25
TBR_MEAN_ increased	4	11
SUV_MAX_ increased	13	37
SUV_MEAN_ increased	24	68
PET_VOL_ 10 (mean)	39 (1–115)	–
PET_VOL_ 60 (mean)	34 (1–101)	–
T1-Gad (mean)	30 (4–89)	–
PET-T1-Gad (mean)	50 (5–131)	–
PET-T1-Gad without tumor bed (mean)	45 (3–129)	–

### Progression-free survival and overall survival

**Fig. 2:**
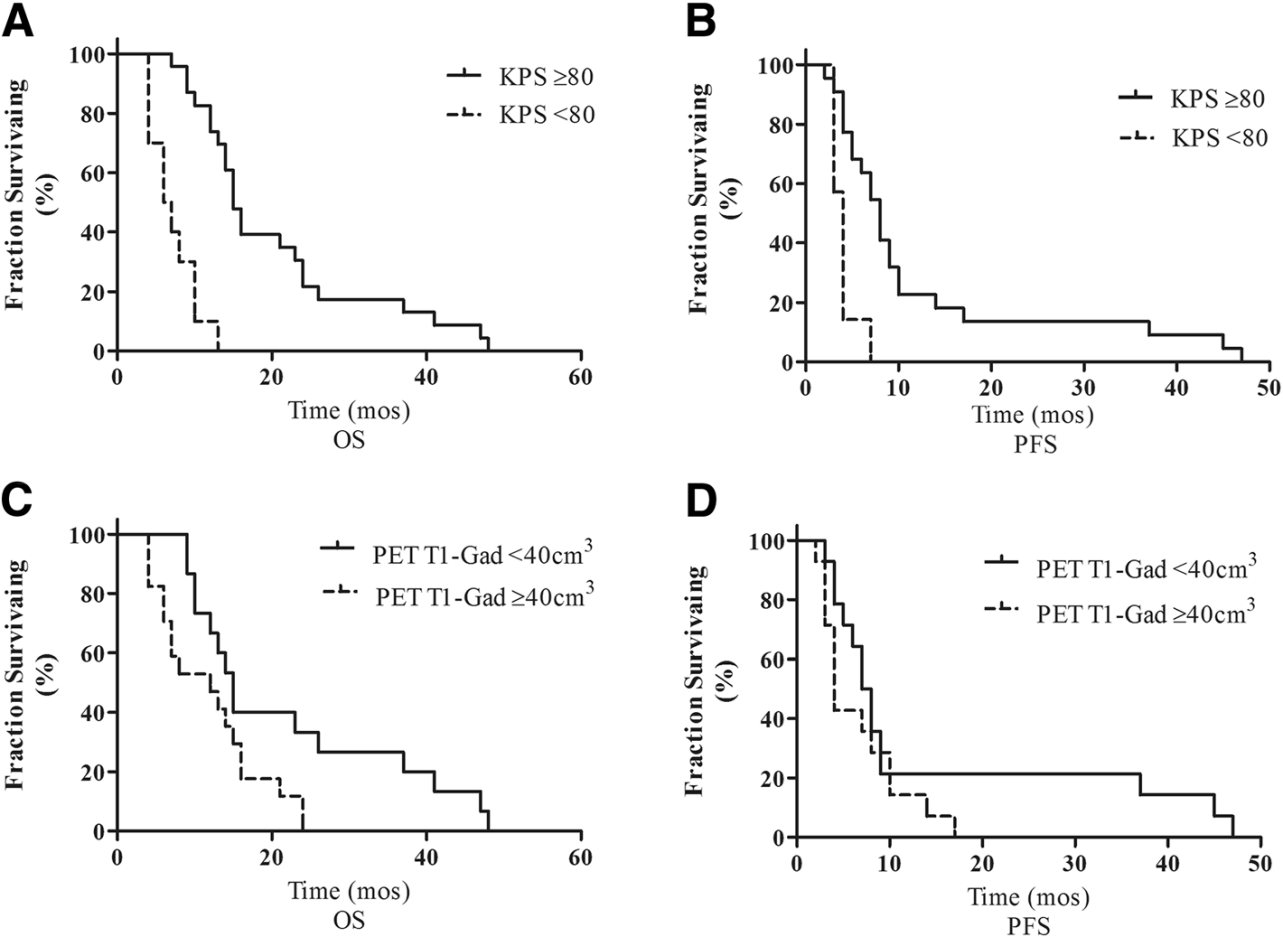
**a** Kaplan-Meier plots of survival versus KPS showing significantly worse survival in patients with KPS values greater than the median (median OS 15 months versus 7 months). **b** Kaplan-Meier plots showing that greater KPS was associated with worse PFS (median PFS 8 versus 4). **c** and **d** PET-T1-Gad volume was associated with worse survival but not PFS, respectively

Better KPS performance status (> 70%) had a favorable impact on PFS (Kaplan-Meier test; HR 0.09, 95% CI 0.02–0.38, *p* = 0.001) and OS (Kaplan-Meier test; HR 0.03, 95% CI 0.007–0.11, *p* = 0.001; Fig. 2a and b and Additional file 1: Table S1), and was correlated with PFS (*p* = 0.007) and OS (*p* < 0.001) as assessed by Spearman’s rank correlations (Additional file 1: Table S2A). Gross total resection had no impact on PFS (*p* = 0.594) or OS (*p* = 0.22) (Additional file 1: Table S2B). Other significant parameters are presented in Additional file 1: Table S1.

There were no statistically significant relationships between PFS and the quantitative imaging parameters in the univariate analysis. However, for OS, there were significant and negative Spearman’s correlations with age, PET_VOL_ 10, PET_VOL_ 60, T1-Gad, PET-T1-Gad, and PET-T1-Gad without tumor bed (Table 2). OS was significantly longer for the group without SVZ involvement (Mann-Whitney test; mean OS 14.1 vs. 18.8 months, median OS 10 vs. 15 months, *p* = 0.021, Table 3), but tumor volumes were significantly smaller in this group (PET-T1-Gad mean volume in SVZ- vs. SVZ+ 35.9 cm^3^ vs. 64.2cm^3^, *p* = 0.004).

**Table 2:** Univariate analysis of patient survival related to selected quantitative imaging factors (Spearman’s rank test)

Parameter	PFS		OS	
	R	*p*	R	*p*
Age	0.130	0.502	−0.375	**0.032**
PET_VOL_10	−0.145	0.454	−0.374	**0.032**
PET_VOL_60	−0.220	0.251	−0.423	**0.014**
PET_VOL_diff	0.355	0.059	0.161	0.372
T1-Gad	−0.293	0.131	−0.441	**0.011**
PET T1-Gad	−0.198	0.312	−0.443	**0.011**
PET-T1 Gad w/o tumor bed	−0.205	0.295	−0.447	**0.010**
TBR_MEAN_ DIFF	0.159	0.418	0.012	0.949
TBR_MAX_ DIFF	0.237	0.225	0.304	0.091
SUV_MEAN_ DIFF	−0.002	0.993	−0.317	0.073
SUV_MAX_ DIFF	0.254	0.183	0.130	0.471
SUV_MAX_10	0.363	0.058	0.184	0.314
SUV_MEAN_ 10	0.021	0.913	−0.163	0.364
TBR_MAX_10	0.300	0.121	0.300	0.095
TBR_MEAN_10	0.152	0.439	0.058	0.753
SUV_MAX_60	0.100	0.604	−0.045	0.803
SUV_MEAN_60	0.081	0.677	−0.069	0.701
TBR_MAX_60	0.140	0.477	0.049	0.790
TBR_MEAN_60	0.041	0.838	0.055	0.767

**Table 3:** Analysis of patient survival and imaging parameters in relation to SVZ involvement (Mann-Whitney test)

Parameter	SVZ involved			SVZ not involved			*p*-values
	Mean	Median	Range	Mean	Median	Range	
PFS	11.7	5.0	3–47	9.1	7.5	2–37	0.650
OS	14.1	10.0	4–48	18.8	15.0	9–41	**0.021**
Age	56.1	58.0	34–73	50.8	48.0	29–72	0.207
PET_VOL_10	49.7	51.1	3.07–115.63	27.0	25.7	1.24–67.93	**0.007**
PET_VOL_60	46.0	50.2	0.79–101.7	21.9	19.4	1.48–60.06	**0.007**
PET VOL DIFF	4.5	4.5	−16.06 - 27,61	5.2	4.4	−3.28 - 20.5	0.832
T1-Gad	38.6	33.5	11.2–89.31	21.5	16.3	4.23–53.48	**0.024**
PET T1-Gad	64.2	62.5	17.8–131.5	35.9	29.9	5.1–103.1	**0.004**
PET-T1Gad w/o tumor bed	59,4	58,6	11.2–129.2	29.7	26.2	2.8: - 86.6	**0.001**
TBR_MEAN_DIFF	0.2	0.2	−0.07 - 0.69	0.3	0.2	−0.09 - 0.8	0.634
TBR_MAX_DIFF	0.3	0.2	−0.43 - 2.33	1.0	0.6	−0.4 - 5.45	0.067
SUV_MEAN_DIFF	−0.1	−0.1	−1.14 - 1.01	0.0	−0.1	− 0.49 - 1.25	0.807
SUV_MAX_DIFF	0.6	0.0	−0.84 - 4.28	0.5	0.6	−1.5 - 3.5	0.832
SUV_MAX_10	3.7	3.3	1.03–10.16	3.1	3.4	1.26–4.79	0.646
SUV_MEAN_10	1.8	1.7	0.8–2.7	1.5	1.5	0.98–2.04	**0.025**
TBR_MAX_10	3.1	2.9	1.92–7.66	3.3	3.0	1.37–6.38	0.734
TBR_MEAN_10	2.5	2.3	2–3.95	2.4	2.3	1.85–3.92	0.518
SUV_MAX_60	3.8	3.4	1.48–8.21	3.0	3.0	1.78–5.36	0.103
SUV_MEAN_60	1.9	1.9	1.18–3.23	1.7	1.7	0.96–2.76	0.207
TBR_MAX_60	2.8	2.5	1.88–5.33	2.3	2.2	0.93–3.2	0.067
TBR_MEAN_60	2.3	2.2	1.89–3.39	2.1	2.0	1.65–3.12	0.079

Significant variables (KPS, SVZ, PET-T1-Gad) in univariable OS analysis and one parameter representing changes in PET uptake values (*TBR*_*MAX*_*diff*) and previously studied by the same authors [34] were entered into a multivariable model (Table 4). KPS and PET-T1-Gad were associated with OS with a close but non-significant relationship for SVZ. For PFS, only KPS was associated in multivariable analysis.

**Table 4:** Multivariate linear regression analyses of OS or PFS versus age, KPS, SVZ, PET-T1-Gad, and TBR_MAX_ DIFF

Parameter	OS	
	Coefficient	*p*-value
Age	−0.11 (0.50)	0.482
KPS	−14.38 (0.45)	**0.006**
SVZ	8.82 (0.25)	0.091
PET-T1-Gad	−0.142 (0.059)	**0.047**
TBR_MAX_ DIFF	0.72 (0.13)	0.680
R^2^ = 0.406		
Parameter	PFS	
	Coefficient	*p*
Age	0.16 (0.19)	0.414
KPS	−13.80 (5.86)	**0.028**
SVZ	10.27 (5.56)	0.078
PET T1 Gad	−0.112 (0.08)	0.194
TBR_MAX_ DIFF	−0.96 (1.92)	0.62
R^2^ = 0.29		

### FET uptake values and kinetics measured in dual time-point assessments

Uptake and kinetic values for the whole group are listed in Table 5, and the kinetic data for patients are presented in Additional file 1: Table S3. In all cases, uptake was above the threshold of 1.6 x mean background. Kinetic analysis was available for 34/35 patients. The majority of GBMs had decreased kinetics measured according to *TBR*_*MEAN*_ *diff* and *TBR*_*MAX*_ *diff* parameters. Kinetic parameters measured quantitatively were not associated with survival (Table 2).

**Table 5:** dtp FET PET parameters in glioblastoma

Parameter	Mean	Median	Range
TBR_MEAN_ DIFF	0.2	0.2	−0.09 – 0.8
TBR_MAX_ DIFF	0.6	0.4	−0.43 – 5.45
SUV_MEAN_ DIFF	−0.1	−0.1	−1.14 – 1.25
SUV_MAX_ DIFF	0.6	0.4	−1.5 – 4.28
SUV_MAX_10	3.4	3.4	1.03–10.16
SUV_MEAN_10	1.6	1.6	0.8–2.7
TBR_MAX_10	3.2	2.9	1.37–7.66
TBR_MEAN_10	2.4	2.3	1.85–3.95
SUV_MAX_60	3.4	3.2	1.48–8.21
SUV_MEAN_60	1.8	1.8	0.96–3.23
TBR_MAX_60	2.6	2.4	0.93–5.33
TBR_MEAN_60	2.2	2.1	1.65–3.39

### FET and SVZ infiltration

SVZ infiltration was present in MRI scans from 18 patients and, by adding dtp FET PET data, three further cases of SVZ infiltration could be defined (21/35; 60%, *p* = 0.25). MRI-based, PET-based, or fused volumes differed significantly when there was SVZ involvement (Additional file 1: Table S5). The most significant difference was for mean PET-T1-Gad without tumor bed (59 cm^3^ in SVZ-positive tumors and 29.7 cm^3^ in SVZ-negative tumors; *p* = 0.001). TBR_MAX_, TBR_MEAN_, and kinetic parameters were nearly identical in both groups. EPE and quantitative parameters above median were not additional negative factors when combined with SVZ (Additional file 1: Table S4).

## Discussion

Here we show that pre-irradiation tumor volumes have a prognostic impact in GBM. Of the analyzed volumes, fused dtp FET PET for T1-Gad-based volume without the tumor bed was the most powerful predictor and may therefore be of value for radiation treatment planning. SVZ involvement, KPS performance status, and age but not the tumor-to-brain uptake ratios or FET kinetics measured by dtp PET/CT were prognostic in univariate analysis. However, the presence or absence of SVZ was associated with higher PET/MR tumor volumes, so this association was no longer significant in multivariate analysis.

Tumors are known to differ in shape and size when defined 10 and 60 min post FET injection and corresponded with the site of recurrence [28]. The current study shows that dtp FET PET parameters does not provides additional information as a prognostic imaging biomarker, although we note that FET PET performed after irradiation treatment response has previously been shown to be a marker of both PFS and OS [9].

Current PET tracers provide additional prognostic value in GBM [9, 35–38]. However, amino acids and FET specifically are most commonly used for PET due to low uptake into inflammatory tissues, high stability, and longer half-life of 18F-FET [39].

Tumor volumes are vulnerable to the FET PET acquisition method. Tumor-to-brain ratios of 1.6 or greater determine the FET tumor volume and depend on the time of measurement, spatial resolution of the PET scans, and image processing [20]. The most commonly used method represents a summation of dynamic PET scans and a single static scan 20–40 min post FET injection. Pre-irradiation tumor volumes defined on static PET have been shown to be prognostic [9]. However, in high-grade gliomas, there can be increased tracer uptake at earlier time frames [26, 40], so tumor volume might be underestimated in the standard 20–40-min scan frequently taken in static PET-based radiation treatment planning [26]. However, in our group, 25% of patients had different uptake kinetics that may underestimate tumor volumes when categorized only on early (5–15 min p.i.) acquisition. The largest study to date on the topic reported a correlation between PET volumes and OS based on dynamic PET results [38]. However, dynamic FET PET is more time-consuming and costly, requiring 40–50 min of scanning time [20], which may be too long to patients to tolerate the thermoplastic mask.

Grosu et al. [41] reported that gross tumor volumes were not significantly different when measured by L-[*methyl*-^11^C]-methionine (MET) PET or FET PET by static acquisition. However, FET PET-based volumes depend on the time of uptake measurement, which may have limited this comparison. Moreover, the different uptake kinetics (also known as the time-activity curve (TAC)) is a feature of FET not observed with MET [20].

Uptake kinetics have been shown to be prognostic in more aggressive low-grade [22] and WHO III [21] gliomas. Further, in a study of selected patients, uptake kinetics had an impact on prognosis [23]. We could not confirm this finding here, perhaps due to the smaller group size, non-selected cohort, or different method of PET acquisition.

With respect to the impact of SVZ invasion on GBM prognosis, our results are consistent with other studies [8, 14, 33]. However, to our knowledge, this is the first report that FET-PET-detected infiltration of the surrounding brain is larger in SVZ areas then in other locations. These large FET uptake volumes may, to some extent, explain worse outcomes for patients with SVZ invasion, which is typically explained in pre-clinical studies by the “neural stem cell niche” concept. Moreover, tumor not defined as infiltrating the SVZ on MRI may actually extend into this area when defined by dtp FET PET. Further, the frequency of SVZ involvement not shown on MRI but present in dtp FET PET is unknown. Here, this frequency was not significantly increased, but this was simply due to the larger volumes defined by dtp FET PET. It could be of value to target SVZ areas with modified or higher than routine radiation doses in future research studies.

The maximal safe resection of contrast-enhancing tumors is the mainstay of treatment for newly diagnosed GBM. Although extensively studied, the prognostic value of partial resection remains controversial, but the benefit of gross total resection in association with survival has been established [42]. This, however, was not seen in our study. Other factors such as *MGMT* methylation have a substantial impact on prognosis and, in combination with the small number of events, could impact on the lack of effect of gross total removal on survival. Here, 24 patients underwent gross total resection as defined by MRI; interestingly, all 35 patients had pathological uptake values in the surrounding tumor bed. This suggests that when the aim is to remove a contrast enhanced portion of a tumor, partial rather than gross tumor removal is the actual result. A recent large retrospective analysis showed that the additional removal of a significant portion of the FLAIR-abnormal region was associated with better survival [43]. Further, PET-based tumor removal may prolong survival in patients with high-grade gliomas [10]. Our study supports the concept that pre-radiation tumor volumes, especially when defined by PET rather than contrast-enhanced tumor removal, influences prognosis [9, 44]. The prognostic value of PET-based volume was also recently reported in a case of re-irradiation [45]. Nevertheless, performance status post-surgery remains the most important clinical marker of treatment outcome.

The dtp FET PET methodology used here and that published by Lohmann et al. [27] are different (20–40 p.i. and 70–90 p.i. in [27] and 5–15 p.i. and 50–60 p.i. here). The frequently recommended acquisition (20–40 p.i.) is not one of the two time points, which might have influenced the results (the threshold of 1.6 of the background has been validated on 20–40 p.i. static images). However, this might explain why 25% of GBMs showed decreased uptake (the maximal uptake could have been obtained in the period 20–40 min p.i., and thus missed by the choice of timepoints).

The main limitations of this study are the post hoc analysis of SVZ infiltration, manual measurement of tumor volumes that may influence results, and the relatively small number of patients. The lack of known *MGMT* promoter methylation status may also be regarded as limiting, but this was not standard care during recruitment. However, it has been shown that the FET PET volumes are independent of *MGMT* methylation status [38]. This study is also strengthened by its prospective nature, no pre-selection of patients, and confirmation of the exact date of death.

## Conclusions

FET PET-based tumor volumes may be useful for predicting a worse prognosis in glioblastoma patients. Although the presence of SVZ infiltration is linked to higher PET/MRI-based tumor volumes, the independent value of dtp FET PET parameters and SVZ infiltration as prognostic markers pre-irradiation has not been confirmed.

## Additional file

Additional file 1: **Table S1.** Univariate analysis of patient survival related to selected factors. The cut-off for the quantitative parameters used (age, PET_VOL_ 10. PET_VOL_ 60. T1-Gad, T1-Gad with tumor bed) was the median index for the entire group determined separately for each indicator. **Table S2.** Spearman’s rank correlations of KPS score (A) (KPS 100–80 – KPS 1, KPS < 80 – KPS 2) and extend of resection (B) (Gross total resection - GTR 1, subtotal resection or biopsy - GTR 0) for overall survival (OS) and progression free survival (PFS). **Table S3.** Kinetic data for all patients. **Table S4.** Combined Kaplan Meier analysis of SVZ with other imaging parameters (above or below median value for quantitative results) for overall survival (OS) and progression free survival (PFS). (DOCX 31 kb)

## Abbreviations

dtp: Dual-time point
FET: O-(2-[^18^F]fluoroethyl)-L-tyrosine
GBM: Glioblastoma multiforme
GPA: Grading prognostic assessment
HGGs: High-grade gliomas
KPS: Karnofsky performance status
LGGs: Low-grade gliomas
MRI: Magnetic resonance imaging
OS: Overall survival
PET: Positron-emission tomography
PFS: Progression-free survival
SUV: Standardized uptake value
SVZ: Subventricular zone
TBR: Tumor-to-brain ratio
VOI: Volumes of interest
