# Supplementary material for: Prognostic value of subventricular zone involvement in relation to tumor volumes defined by fused MRI and O-(2-[18F]fluoroethyl)-L-tyrosine (FET) PET imaging in glioblastoma multiforme
Source: Radiat Oncol. 2019 Mar 4;14:37. doi: 10.1186/s13014-019-1241-0 (PMC6398237; doi:10.1186/s13014-019-1241-0)
Supplement: Supplementary file 1 — Table S1. Univariate analysis of patient survival related to selected factors. The cut-off for the quantitative parameters used (age, PETVOL 10. PETVOL 60. T1-Gad, T1-Gad with tumor bed) was the median index for the entire group determined separately for each indicator. Table S2. Spearman’s rank correlations of KPS score (A) (KPS 100–80 – KPS 1, KPS < 80 – KPS 2) and extend of resection (B) (Gross total resection - GTR 1, subtotal resection or biopsy - GTR 0) for overall survival (OS) and progression free survival (PFS). Table S3. Kinetic data for all patients. Table S4. Combined Kaplan Meier analysis of SVZ with other imaging parameters (above or below median value for quantitative results) for overall survival (OS) and progression free survival (PFS). (DOCX 31 kb) [file 13014_2019_1241_MOESM1_ESM.docx]

**Additional file 1**

**Table S1** Univariate analysis of patient survival related to selected factors. The cut-off for the quantitative parameters used (age, PET_VOL_ 10. PET_VOL_ 60. T1-Gad, T1-Gad with tumor bed) was the median index for the entire group determined separately for each indicator.

|  | **OS** | | | **PFS** | | |
| --- | --- | --- | --- | --- | --- | --- |
| **Parameter** | ***p*** | ***HR*** | ***95% Cl*** | ***P*** | ***HR*** | ***95% CI*** |
| Gross total resection on MRI | 0.409 | 1.38 | 0.64-2.95 | 0.677 | 1.20 | 0.51-2.84 |
| Age (<53 years vs. ≥53 years) | 0.435 | 0.74 | 0.35-1.57 | 0.344 | 1.51 | 0.64-3.54 |
| **KPS performance status (<80 vs. ≥80)** | **<0.001** | 0.03 | 0.007-0.11 | **0.001** | 0.09 | 0.02-0.38 |
| SVZ | 0.302 | 0.67 | 0.32-1.43 | 0.751 | 1.14 | 0.50-2.60 |
| EPE | 0.739 | 1.14 | 0.528 | 0.552 | 1.32 | 0.53-3.26 |
| **PET_VOL_10** | **0.024** | 0.40 | 0.18-0.89 | 0.297 | 0.64 | 0.27-1.49 |
| PET_VOL_60 | 0.373 | 0.72 | 0.34-1.49 | 0.815 | 0.909 | 0.41-2.03 |
| **T1-Gad** | **0.015** | 1.36 | 0.860 | 0.062 | 0.44 | 0.19-1.04 |
| **PET-T1-Gad** | **0.023** | 0.40 | 0.18-0.88 | 0.174 | 0.55 | 0.23-1.30 |
| **PET-T1-Gad (larger of PET volumes + T1-Gad without tumor bed)** | **0.023** | 0.40 | 0.18-0.88 | 0.174 | 0.55 | 0.23-1.30 |
| Local/distant progression | 0.725 | 0.85 | 0.34-2.10 | 0.248 | 0.57 | 0.22-1.48 |

**Table S2**  Spearman’s rank correlations of KPS score (A) (KPS 100-80 – KPS 1, KPS<80 – KPS 2 ) and extend of resection (B) (Gross total resection - GTR 1, subtotal resection or biopsy - GTR 0) for overall survival (OS) and progression free survival (PFS).

A

| Parameter | KPS 1 | | | KPS 2 | | | p values |
| --- | --- | --- | --- | --- | --- | --- | --- |
|  | Mean | Median | Range | Mean | Media | Range |  |
| PFS | 12.2 | 8.0 | 2 : 47 | 4.0 | 4.0 | 3 : 7 | **0.007** |
| OS | 20.3 | 15.0 | 7 : 48 | 7.2 | 6.5 | 4 : 13 | **<0.001** |

B

| Parameter | GTR 0 | | | GTR 1 | | | p values |
| --- | --- | --- | --- | --- | --- | --- | --- |
|  | Mean | Median | Range | Mean | Median | Range |  |
| PFS | 10.0 | 6.5 | 2 : 45 | 10.8 | 7.0 | 4 : 47 | 0.594 |
| OS | 14.79 | 12.5 | 4 : 48 | 20.6 | 15.0 | 9 : 47 | 0.222 |

**Table S3** Kinetic data for all patients.

| **Patient**  **number** | **TBR_MEAN_diff** | **TBR_MAX_diff** | **SUV_MEAN_diff** | **SUV_MAX_diff** | **SUV_MAX_10** | **SUV _MEAN_10** | **TBR_MAX_10** | **TBR_MEAN_10** | **SUV_MAX_60** | **SUV_MEAN_60** |
| --- | --- | --- | --- | --- | --- | --- | --- | --- | --- | --- |
| 1 | 0.56 | 0.93 | 0.21 | 101 | 5.28 | 2.02 | 4.19 | 3.95 | 4.27 | 181 |
| 2 | 0.8 | 3.18 | 0.05 | 126 | 4.79 | 186 | 6.38 | 3.92 | 3.53 | 181 |
| 3 | 0.69 | 0.93 | 0.29 | 0.91 | 4.72 | 2.41 | 3.21 | 3.13 | 3.81 | 2.12 |
| 4 | 0.68 | 133 | 0.05 | 105 | 3.85 | 174 | 4.18 | 2.9 | 2.8 | 169 |
| 5 | 0.21 | -0.33 | 0.1 | 0.26 | 4.62 | 19 | 3.64 | 2.71 | 4.36 | 18 |
| 6 | 0.24 | -0.28 | 0.01 | -0.6 | 3.22 | 165 | 2.05 | 2.17 | 3.82 | 164 |
| 7 | 0.33 | 0.82 | -0.06 | 0.65 | 3.81 | 163 | 2.95 | 2.36 | 3.16 | 169 |
| 8 | -0.09 | 0.4 | -0.38 | -15 | 2.56 | 176 | 2.27 | 191 | 4.06 | 2.14 |
| 9 | 0.09 | 0.17 | -0.31 | -0.14 | 3.36 | 169 | 2.09 | 2.04 | 3.5 | 2 |
| 10 | -0.01 | -0.11 | -0.49 | -0.84 | 2.51 | 145 | 2.89 | 2.07 | 3.35 | 194 |
| 11 | 0.11 | -0.39 | -0.09 | 0.05 | 2.66 | 177 | 194 | 2.27 | 2.61 | 186 |
| 12 | 0.08 | -0.07 | -0.13 | -0.3 | 2.51 | 132 | 2.49 | 2.1 | 2.81 | 145 |
| 13 | 0.04 | -0.43 | -0.14 | -0.08 | 2.79 | 178 | 192 | 2 | 2.87 | 192 |
| 14 | 0.06 | 0.26 | 0.08 | -0.11 | 3.38 | 2.24 | 2.75 | 2.22 | 3.49 | 2.16 |
| 15 | 0.12 | 0.56 | -0.03 | 0.67 | 3.37 | 142 | 2.63 | 2.25 | 2.7 | 145 |
| 16 | 0.64 | 2.33 | 101 | 195 | 10.16 | 2.7 | 7.66 | 3.94 | 8.21 | 169 |
| 17 | 0.06 | 0.45 | -0.18 | 0.03 | 3.04 | 172 | 2.55 | 2.02 | 3.01 | 19 |
| 18 | 0.18 | 0.15 | -0.13 | 0.57 | 3.76 | 189 | 2.03 | 2.07 | 3.19 | 2.02 |
| 19 | -0.07 | -0.4 | -114 | 4.28 | 4.28 | 2.11 | 3.4 | 2.25 | 7.45 | 3.23 |
| 20 | 0.16 | 0.14 | -0.28 | 2.07 | 2.07 | 13 | 3.14 | 2.36 | 2.97 | 164 |
| 21 | 0.1 | 0.39 | -0.14 | 182 | 182 | 113 | 2.3 | 2.03 | 197 | 123 |
| 22 | 0.17 | 0.61 | 125 | 0 |  | 125 | 3.47 | 2.25 | 3.27 | 166 |
| 23 | 0.32 | 0.11 | 104 | 3.5 | 3.5 | 2.04 | 3.2 | 2.66 | 5.36 | 2.76 |
| 24 | 0.42 | 115 | -0.16 | 3.05 | 3.05 | 142 | 3.46 | 2.64 | 2.64 | 155 |
| 25 | 0.37 | 191 | -0.25 | 0.35 | 2.74 | 124 | 4.15 | 2.53 | 2.39 | 149 |
| 26 | -0.02 | -0.4 | -0.44 | -0.69 | 126 | 0.98 | 137 | 185 | 195 | 142 |
| 27 | 0.52 | 5.45 | -0.19 | 16 | 3.38 | 1 | 6.38 | 2.44 | 178 | 119 |
| 28 | 0.1 | -0.22 | -0.42 | -0.54 | 192 | 117 | 2.02 | 2.02 | 2.46 | 159 |
| 29 | 0.08 | 0.3 | -0.46 | -0.3 | 2.66 | 151 | 2.42 | 196 | 2.96 | 197 |
| 30 | 0.13 | 0.24 | -0.42 | -0.48 | 3.4 | 179 | 2.74 | 2.29 | 3.88 | 2.21 |
| 31 | 0.55 | 0.58 | -0.02 | 0.38 | 3.71 | 156 | 3.14 | 2.2 | 3.33 | 158 |
| 32 | 0.02 | 0.88 | -0.49 | 0.61 | 3.98 | 155 | 2.65 | 185 | 3.37 | 2.04 |
| 33 | 0.35 | 0.61 | -0.27 | -0.18 | 3.83 | 161 | 3.87 | 2.52 | 4.01 | 188 |
| 34 | 0.42 | 0.61 | 0.14 | 0.66 | 2.95 | 11 | 3.47 | 2.56 | 2.29 | 0.96 |
| 35 |  |  | -0.28 | -0.45 | 103 | 0.8 |  |  | 148 | 118 |

**Table S4**  Combined Kaplan Meier analysis of SVZ with other imaging parameters (above or below median value for quantitative results) for overall survival (OS) and progression free survival (PFS).

| Parameter | OS  p | PFS  p |
| --- | --- | --- |
| Kinetics  (decreased vs. increased) | 0.071 | 0.197 |
| EPE | 0.662 | 0.902 |
| PET_VOL_10 | 0.205 | 0.799 |
| PET_VOL_60 | 0.170 | 0.952 |
| PET_VOL_diff | 0.239 | 0.499 |
| SUV_MAX_10 | 0.686 | 0.763 |
| SUV_MEAN_10 | 0.797 | 0.766 |
| TBR_MAX_10 | 0.499 | 0.675 |
| TBR_MEAN_10 | 0.407 | 0.578 |
| SUV_MAX_60 | 0.825 | 0.984 |
| SUV_MEAN_60 | 0.654 | 0.156 |
| TBR_MAX_60 | 0.528 | 0.953 |
| TBR_MEAN_60 | 0.721 | 0.839 |
